# Enhancing saline stress tolerance in soybean seedlings through optimal NH_4_^+^/NO_3_^−^ ratios: a coordinated regulation of ions, hormones, and antioxidant potential

**DOI:** 10.1186/s12870-024-05294-z

**Published:** 2024-06-18

**Authors:** Javaria Noor, Izhar Ahmad, Abd Ullah, Babar Iqbal, Shazma Anwar, Arshad Jalal, Mohammad K. Okla, Ibrahim A. Alaraidh, Hamada Abdelgawad, Shah Fahad

**Affiliations:** 1 Department of Botany, Islamia College Peshawar, Peshawar, Khyber Pakhtunkhwa Pakistan; 2 Xinjiang Key Laboratory of Desert Plant Roots Ecology and Vegetation Restoration, Xinjiang Institute of Ecology and Geography, Chinese Academy of Sciences, Urumqi, 830011 People’s Republic of China; 3 School of Environment and Safety Engineering, Jiangsu University, Zhenjiang, 212013 People’s Republic of China; 4 Department of Agronomy, Faculty of Crop Production Sciences, The University of Agriculture, Peshawar, 25000 Pakistan; 5 School of Engineering, Department of Plant Health, Rural Engineering and Soils, São Paulo State University - UNESP-FEIS, Ilha Solteira, São Paulo, 15385-000 Brazil; 6 Botany and Microbiology Department, College of Science, King Saud University, P.O. Box 2455, Riyadh, 11451 Saudi Arabia; 7 Integrated Molecular Plant Physiology Research, Department of Biology, University of Antwerp, Antwerp, 2020 Belgium; 8 Department of Agronomy, Abdul Wali Khan University Mardan, Mardan, Khyber Pakhtunkhwa 23200 Pakistan

**Keywords:** Abiotic stress, Physiological responses, Salinity resistance, Nutrients management, Nitrogen form

## Abstract

**Background:**

Nitrogen (N) availability is crucial in regulating plants’ abiotic stress resistance, particularly at the seedling stage. Nevertheless, plant responses to N under salinity conditions may vary depending on the soil’s NH_4_^+^ to NO_3_^−^ ratio.

**Methods:**

In this study, we investigated the effects of different NH_4_^+^:NO_3_^−^ ratios (100/0, 0/100, 25/75, 50/50, and 75/25) on the growth and physio-biochemical responses of soybean seedlings grown under controlled and saline stress conditions (0-, 50-, and 100-mM L^− 1^ NaCl and Na_2_SO_4_, at a 1:1 molar ratio).

**Results:**

We observed that shoot length, root length, and leaf-stem-root dry weight decreased significantly with increased saline stress levels compared to control. Moreover, there was a significant accumulation of Na^+^, Cl^−^, hydrogen peroxide (H_2_O_2_), and malondialdehyde (MDA) but impaired ascorbate-glutathione pools (AsA-GSH). They also displayed lower photosynthetic pigments (chlorophyll-a and chlorophyll-b), K^+^ ion, K^+^/Na^+^ ratio, and weakened O_2_^•−^-H_2_O_2_-scavenging enzymes such as superoxide dismutase, catalase, peroxidase, monodehydroascorbate reductase, glutathione reductase under both saline stress levels, while reduced ascorbate peroxidase, and dehydroascorbate reductase under 100-mM stress, demonstrating their sensitivity to a saline environment. Moreover, the concentrations of proline, glycine betaine, total phenolic, flavonoids, and abscisic acid increased under both stresses compared to the control. They also exhibited lower indole acetic acid, gibberellic acid, cytokinins, and zeatine riboside, which may account for their reduced biomass. However, NH_4_^+^:NO_3_^−^ ratios caused a differential response to alleviate saline stress toxicity. Soybean seedlings supplemented with optimal ratios of NH_4_^+^:NO_3_^−^ (T3 = 25:75 and T = 4 50:50) displayed lower Na^+^ and Cl^−^ and ABA but improved K^+^ and K^+^/Na^+^, pigments, growth hormones, and biomass compared to higher NH_4_^+^:NO_3_^−^ ratios. They also exhibited higher O_2_^•−^-H_2_O_2_-scavenging enzymes and optimized H_2_O_2_, MDA, and AsA-GSH pools status in favor of the higher biomass of seedlings.

**Conclusions:**

In summary, the NH_4_^+^ and NO_3_^−^ ratios followed the order of 50:50 > 25:75 > 0:100 > 75:25 > 100:0 for regulating the morpho-physio-biochemical responses in seedlings under SS conditions. Accordingly, we suggest that applying optimal ratios of NH_4_^+^ and NO_3_^−^ (25/75 and 50:50) can improve the resistance of soybean seedlings grown in saline conditions.

## Background

Soil salinization poses a significant threat to global agriculture, affecting 3600 million hectares (Mha) out of 5200 Mha of arable land, resulting in annual losses of USD 27.5 billion [1]. Due to climate change and improper agricultural practices, soil salinity is expected to increase, rendering soils non-fertile and unusable for agricultural purposes. Alkaline salt stress is caused by alkaline salts (NaHCO_3_ and Na_2_CO_3_), while saline stress is caused by neutral salts or saline salts (NaCl and Na_2_SO_4_). These two salt stresses affect approximately 932 Mha worldwide [2]. Typically, soil salinity reduces photosynthetic ability, decreases nutrient uptake, destabilizes membranes, damages antioxidant defense mechanisms, impairs metabolism, and compromises cellular membranes [3, 4]. Plants employ various mechanisms to mitigate salinity damage, including the (a) accumulation of osmolytes, (b) exclusion of or compartmentalization of toxic salt ions (Na^+^ and Cl^−^), and (c) upregulation of antioxidant enzymes and metabolites to counteract excessive reactive oxygen species (ROS) [4–7]. During stress, plants accumulate osmolytes such as proline, soluble sugar, glycine betaine, and amino acids, which assist in maintaining salinity homeostasis and osmotic adjustment. These osmolytes are essential in maintaining water balance, protecting plants from salinity-induced damage, preventing ion toxicity and chlorophyll loss, regulating cell division, stabilizing cellular structures, and scavenging ROS [8, 9].

In addition, salinity increases the accumulation of ROS and deteriorates membrane permeability and structure [10]. In response, plants modulate their antioxidant enzymes including superoxide dismutase (SOD), peroxidase (POD), ascorbate peroxidase (APX), glutathione reductase (GR), glutathione peroxidase (GPX) and polyphenol oxidase (PPO), and monodehydroascorbate reductase (MDHAR), and dehydroascorbate reductase (DHAR)] along with metabolites such as flavonoids, phenols, proline, ascorbate (AsA) and glutathione (GSH)] to counterbalance excessive ROS. Consequently, plant tolerance to salinity stress is associated with increased antioxidant defense mechanisms [6, 11, 12].

Moreover, phytohormones are endogenous signaling molecules directly involved in plants’ physiological and biochemical processes under normal and stressful conditions. These include abscisic acid (ABA), salicylic acid (SA), jasmonic acid (JA), gibberellic acid (GA), indole acetic acid (IAA), cytokinins (CTK), zeatine ribosides (ZR), and brassinosteroids (BRs) [13–15]. There is widespread recognition of the roles of phytohormones in modulating physiological and biochemical processes in plants under salinity stress [14]. Endogenous hormone concentrations may help in predicting the mechanisms of plant tolerance or susceptibility to diverse environmental stress conditions. These hormones stimulate the expression of several proteins under stress [16]. Understanding hormonal responses provides crucial insight into mechanisms underlying adaptations to saline soils. Ion uptake and homeostasis are essential for average plant growth. During salt stress, salt ions accumulate in plant tissues, leading to ion toxicity and inhibiting mineral ions absorption [17–19], impairing cellular metabolism and growth.

Nutrient management under saline conditions may benefit plants and overcome salinity stress, particularly glycophytes [20]. Adequate nitrogen (N) supply can induce tolerance mechanisms in plants by inhibiting Na^+^ and Cl^−^ ions accumulations due to the antagonistic effects of ammonium (NH_4_^+^) and nitrate (NO_3_^−^) [21–23]. The effectiveness of N nutrition in alleviating salt stress varies depending on the applied N form (NH_4_^+^ or NO_3_^−^) in soil environments [24]. Britto and Kronzucker [25] reported that applying N as NH_4_^+^ under saline conditions is economically advantageous compared to NO_3_^−^. However, higher accumulations of NH_4_^+^ in plant tissues may result in toxicity, accelerating detrimental effects of salinity. Additionally, N nutrition in the form of NO_3_^−^ has been reported to contribute to decreased plant growth under salinity due to increased Na^+^ uptake and more significant energy costs for assimilation [26, 27]. Studies suggest applying NH_4_^+^ and NO_3_^−^ enhances crop growth in saline environments [23, 28–31]. However, interactions between N nutrition and salinity vary depending on plant age, salt stress level, duration, and application method. Previous studies on this topic often presented contradictory findings and lacked information on the effect of NH_4_^+^:NO_3_^−^ ratios on soybean seedlings under saline stress conditions.

Cultivated soybean (*Glycine max* (L.) Merr.) is an economically crucial oil-producing crop, accounting for 30% of global edible oil production and 69% of the world’s dietary protein supply [32]. Despite its significance, soybeans are typically salt-sensitive, necessitating improvements in salinity resistance to optimize their utilization of salinized soils [33]. Although the soybean plants obtain some N through a biological process, their seeds are high in protein, resulting in a substantial demand for N. Additionally, soybean seedlings require more N than other crops to grow normally [34]. Consequently, we tested the effects of varying ratios of NH_4_^+^: NO_3_^−^ (N sources) on soybean seedlings under saline stress to enhance their salinity tolerance and to meet their N requirements for better adaptation to salinized soils. This study hypothesized that an optimal ratio of NH_4_^+^ and NO_3_^−^ treatment could reduce the adverse effects of saline stress on soybean seedlings, leading to improved growth and stress tolerance.

Therefore, we investigated (a) the effects of different ratios of NH_4_^+^ and NO_3_^−^ treatments (100/0–0/100-, 25/75-, 50/50-, and 75/25-ratio) on growth parameters (shoot length, root length, and leaf-stem-root dry weight) and physiological responses (ions regulation, osmolytes, phytohormones, ROS production, lipid peroxidation and O_2_^−^-H_2_O_2_-scavinging mechanisms) of soybean seedlings under saline stress levels (SS; 0-, 50, and 100 mM L^− 1^ NaCl and Na_2_SO_4_, at a 1:1 molar ratio). This study will provide insights into the effects of different ratios of NH_4_^+^ and NO_3_^−^ treatments in modulating the physiological mechanism in plants under saline stress.

## Results

### Changes in seedling growth

**Fig. 1:**
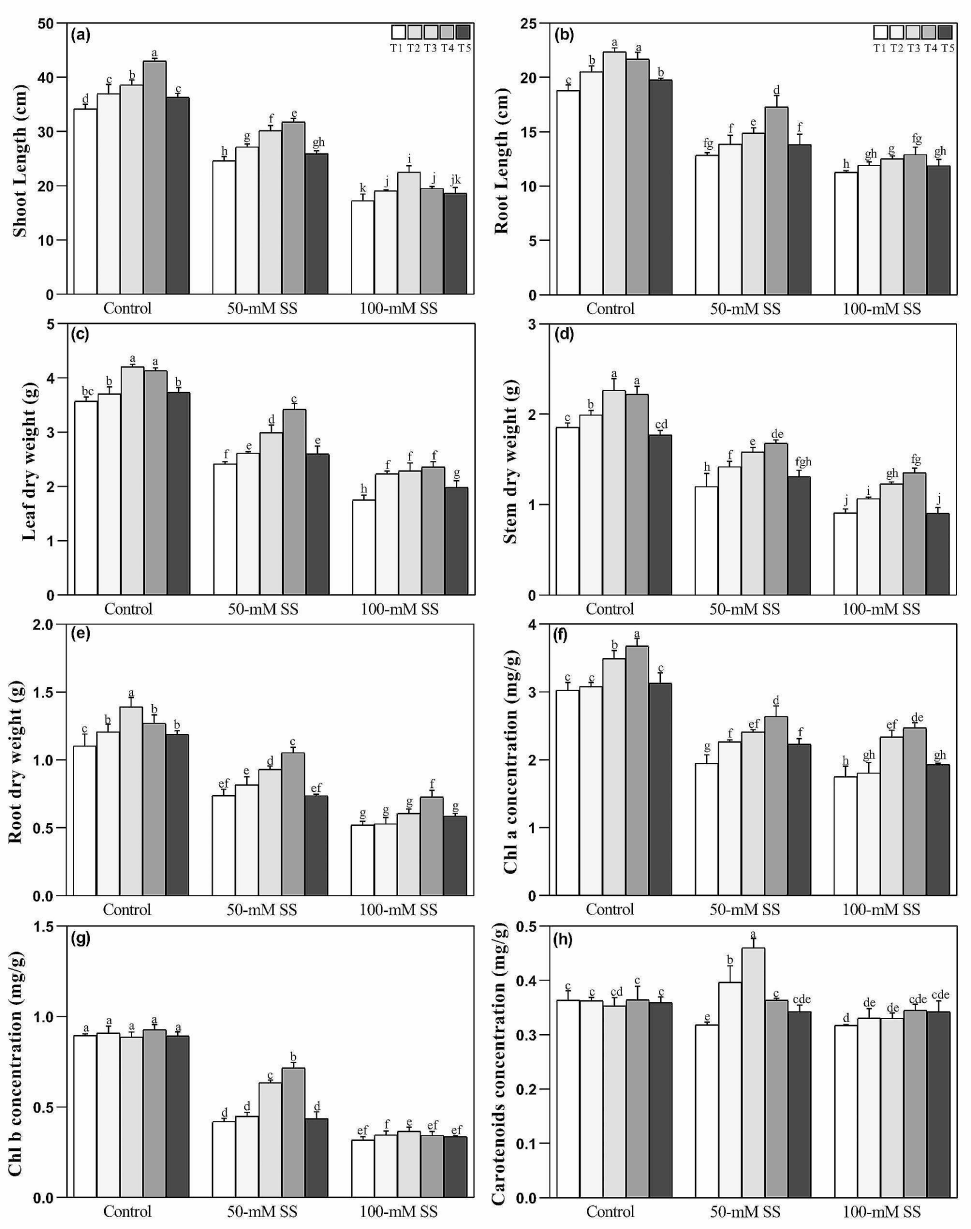
Effect of different ratios of NH_4_^+^:NO_3_^–^ ratio on (**a**) shoot length (**b**) root length (**c**) leaf dry weight, (**d**) stem dry weight, (**e**) root dry weight (**f**) chlorophyll a, (**g**) chlorophyll b, and (**h**) carotenoids in soybeans under controlled and saline stress conditions. Bars represent means ± SD (*n* = 3). Different letters indicate treatment differences at *p* < 0.05 (Duncan’s method)

The growth attributes of soybean seedlings exposed to varying saline stress (SS) and NH_4_^+^:NO_3_^-^ (ammonium: nitrate; AN) ratios exhibited distinct differences compared to seedlings grown under controlled conditions without AN supplementation. As the SS levels increased, there were significant reductions in shoot length, root length, and dry weight of leaves stem and root (Fig. 1a-e). These reductions were more pronounced under 100 mM compared to 50 mM SS. However, applying optimal AN ratios (T3 = 25/75 and T4 = 50/50) led to significant improvements in plant height and biomass of soybean seedlings under both SS levels. The effectiveness of different AN ratios in enhancing salinity stress resistance generally followed the order of 50:50 > 25:75 > 0:100 > 75:25 > 100:0.

Notably, our results depicted that optimum ratios (T3 = 25/75 and T4 = 50/50) of NH_4_^+^/NO_3_^−^ significantly impacted leaf and stem biomass accumulation under controlled conditions and both stress levels (Fig. 1c, d). Additionally, shoot length in control and 50-mM SS wherease root dry weight under 50-mM (Fig. 1a, e) were notably enhanced compared to higher NH_4_^+^/NO_3_^−^ ratios (T1 = 100/0 and T5 = 75/25). Furthermore, the root length of seedlings under control and 50-mM stress conditions significantly increased under T3 and T4 (optimal AN) (Fig. 1b).

### Changes in photosynthetic pigments

Compared to the control group, chlorophyll a, b, and carotenoid concentrations were significantly reduced under both SS levels, regardless of NH_4_^+^/NO_3_^−^ application (Fig. 1f-h). However, optimal NH_4_^+^/NO_3_^−^ ratios (T3 and T4) significantly improved the concentration of chlorophyll a under both stress levels, compared to T1 and T5, while chlorophyll b concentration showed significant improvements under 50-mM stress (Fig. 1f, g).

### Changes in ions accumulation

**Fig. 2:**
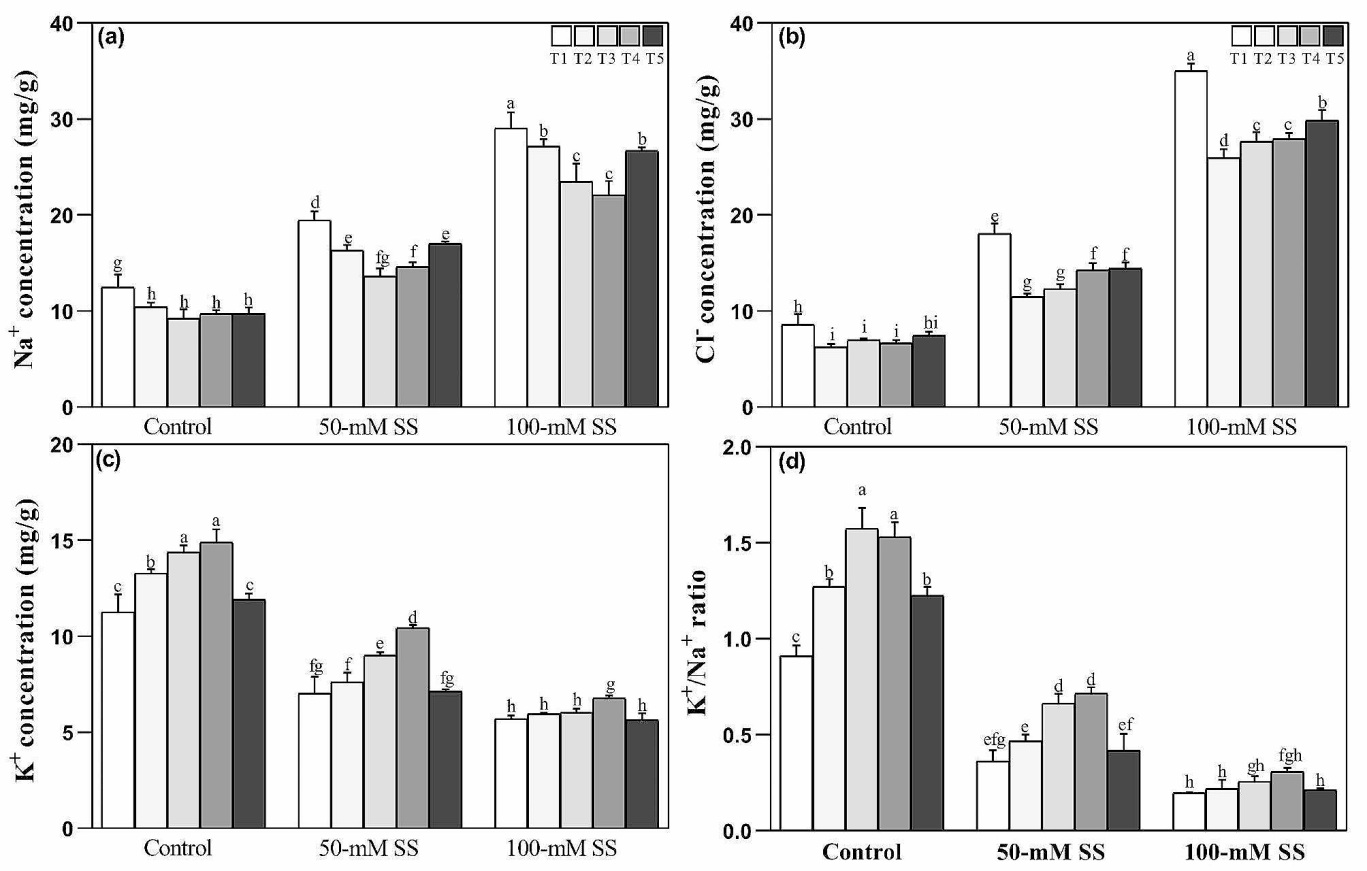
Effect of different ratios of NH_4_^+^:NO_3_^–^ ratio on (**a**) Na^+^ (**b**) Cl^–^ (**c**) K^+^, and (**d**) K^+^/Na^+^, in soybeans under controlled and saline stress conditions. Bars represent means ± SD (*n* = 3). Different letters indicate treatment differences at *p* < 0.05 (Duncan’s method)

Regarding ions accumulations, Na^+^ and Cl^–^ concentrations increased while K^+^ ions decreased, resulting in a K^+^/Na^+^ ratio under both SS levels, irrespective of NH_4_^+^:NO_3_^–^ application (Fig. 2a-d). However, different AN ratios effectively reduced the levels of toxic salt ions and improved the K^+^ concentration and K^+^/Na^+^ ratios. The NH_4_^+^:NO_3_^–^ ratios varied in their effects on salt and mineral ions concentration in soybean seedlings under SS conditions. Optimal NH_4_^+^:NO_3_^–^ ratios (T3 and T4) reduced Na^+^ ion and Cl^–^ ion concentration (Fig. 2a, b) under both stress levels and improved K^+^ levels under 50-mM stress, along with the K^+^/Na^+^ ratio under both stress levels (Fig. 2c, d).

### Responses of oxidative stress indicators and anti-oxidant enzymes

**Fig. 3:**
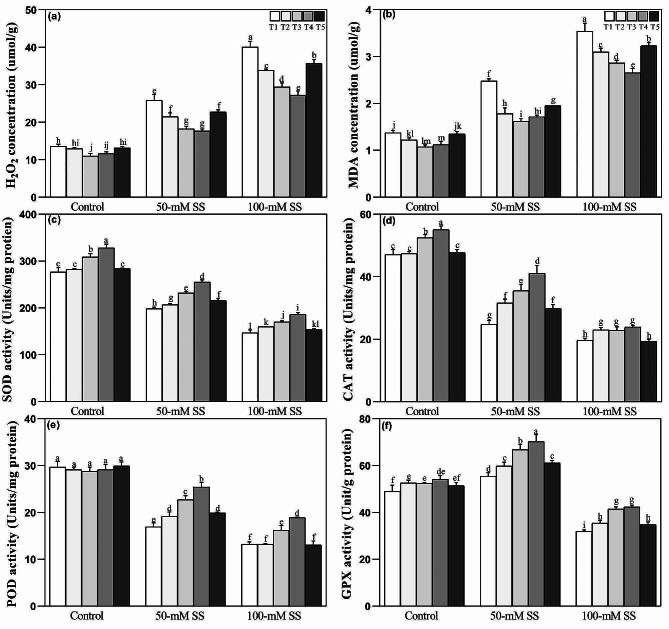
Effect of different ratios of NH_4_^+^:NO_3_^–^ ratio on (**a**) H_2_O_2_ (**b**) MDA (**c**) SOD, (**d**) CAT, (**e**) POD, and (**f**) GPX in soybeans under controlled and saline stress conditions. Bars represent means ± SD (*n* = 3). Different letters indicate treatment differences at *p* < 0.05 (Duncan’s method)

Under both stress levels, oxidative stress biomarkers such as H_2_O_2_ and MDA were up-regulated in the leaves of soybean seedlings compared to control (Fig. 3a, b). However, NH_4_^+^:NO_3_^–^ application significantly reduced their concentrations. Notably, significantly lower H_2_O_2_ and MDA levels were observed under both stress conditions at optimal AN ratios (25/75 > 50/50) compared to T1, T2, and T5 (100/0, 100/0, and 75/25). Additionally, soybean seedlings exposed to SS levels displayed a weakened O_2_^–^ -H_2_O_2_ scavenging mechanism by downregulating the enzymatic activities of SOD, CAT, and POD under both stress levels, and GPX under 100-mM stress, compared to the control (Fig. 3c-f). Although AN supplementation improved their activities, the AN ratios significantly differed in their effects on enhancing anti-oxidant enzymes in SS-treated soybean seedlings. Optimal AN application (T3 = 25/75 and T4 = 50/50) resulted in significantly higher SOD and POD under 50- and 100-mM stress (Fig. 3c, e); CAT under 50-mM, and GPX under 100-mM (Fig. 3d, f), compared to other ratios. Moreover, T3 and T4 also caused significant increments in CAT and SOD under control-treated seedlings. Furthermore, T3 and T4 also caused significant increments in CAT and SOD under control-treated seedlings (Fig. 3c, d).

### Responses of ascorbate-glutathione cycle

**Fig. 4:**
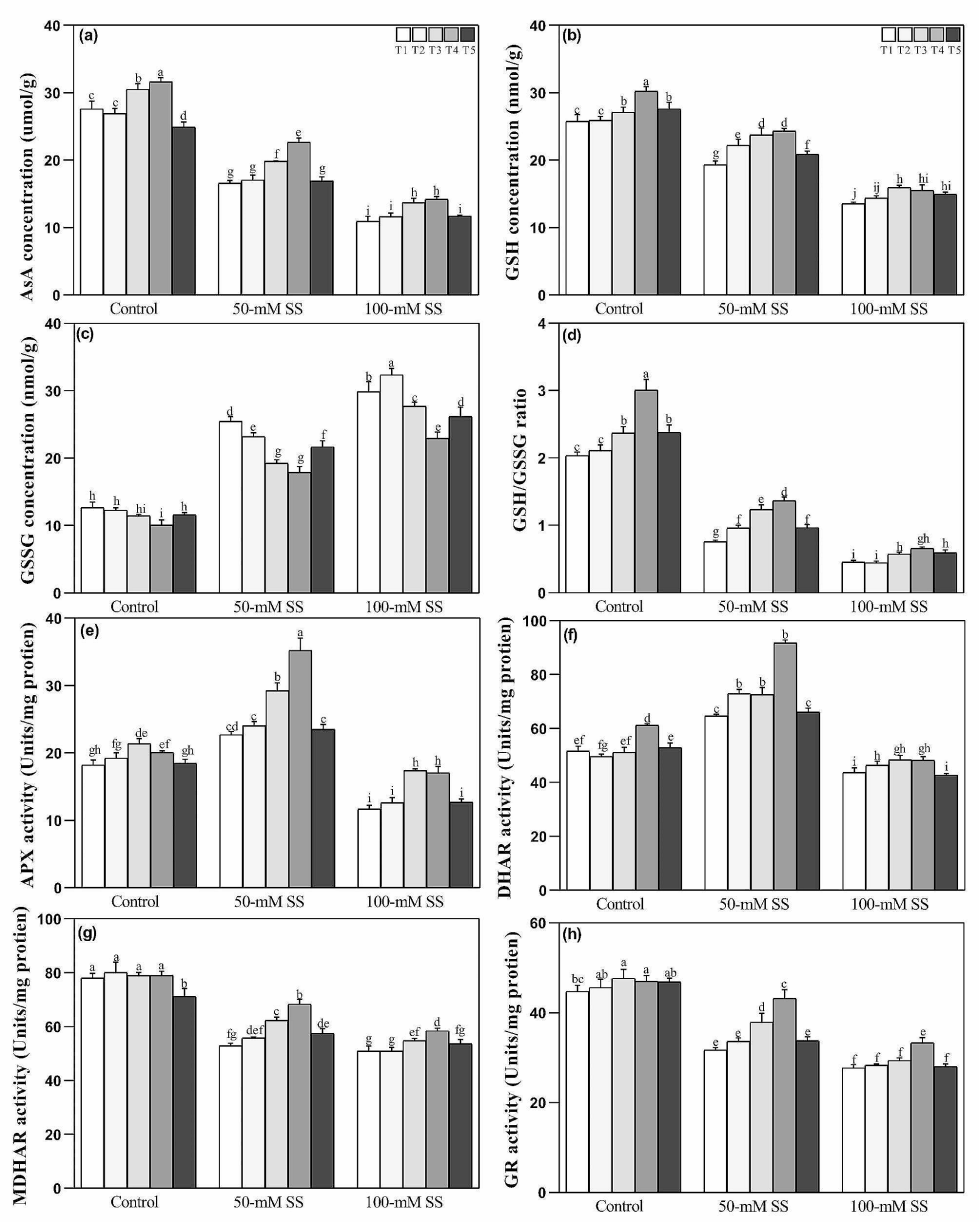
Effect of different ratios of NH_4_^+^:NO_3_^–^ ratio on (**a**) AsA (**b**) GSSG (**c**) GSH, (**d**) GSH/GSSG, (**e**) APX (**f**) DHAR (**g**) MDHAR, and (**h**) GR in soybeans under controlled and saline stress conditions. Bars represent means ± SD (*n* = 3). Different letters indicate treatment differences at *p* < 0.05 (Duncan’s method)

Responses of the ascorbate-glutathione (AsA-GSH) cycle showed that under both stress levels, the AsA) and GSH concentrations decreased. At the same time, those of oxidized glutathione (GSSG) increased (Fig. 4a-c), regardless of AN application. Moreover, soybean seedlings displayed significantly lower GSH/GSSG ratio under both stress levels (Fig. 4d). Among the enzymes of the AsA-GSH cycle, the activities of APX and DHAR enhanced under 50-mM stress but displayed downregulation following 100-mM stress (Fig. 4e, f). Moreover, MDHAR and glutathione reductase (GR) activities decreased under both SS levels, regardless of different ratios of NH_4_^+^/NO_3_^−^ application (Fig. 4g, h). However, AN application stabilized the AsA-GSH cycle by maintaining the AsA-GSH redox state and their metabolizing enzymes. Optimal AN ratios (T3 = 25/75 and T4 = 50/50) caused a significant reduction in GSSG. Still, they improved AsA, APX, and MDHAR under either stress level while enhancing GSH and GSH/GSSG ratio, and the enzymatic activities of DHAR and GR under 50-mM compared to T1 and T5 (100/0 and 75/25). Moreover, under 100-mM stress, the highest activity of GR and the ratio of GSH/GSSG were reported under T3, compared to other NH_4_^+^/NO_3_^−^ ratios (Fig. 4a-h).

### Changes in phytohormone responses

**Fig. 5:**
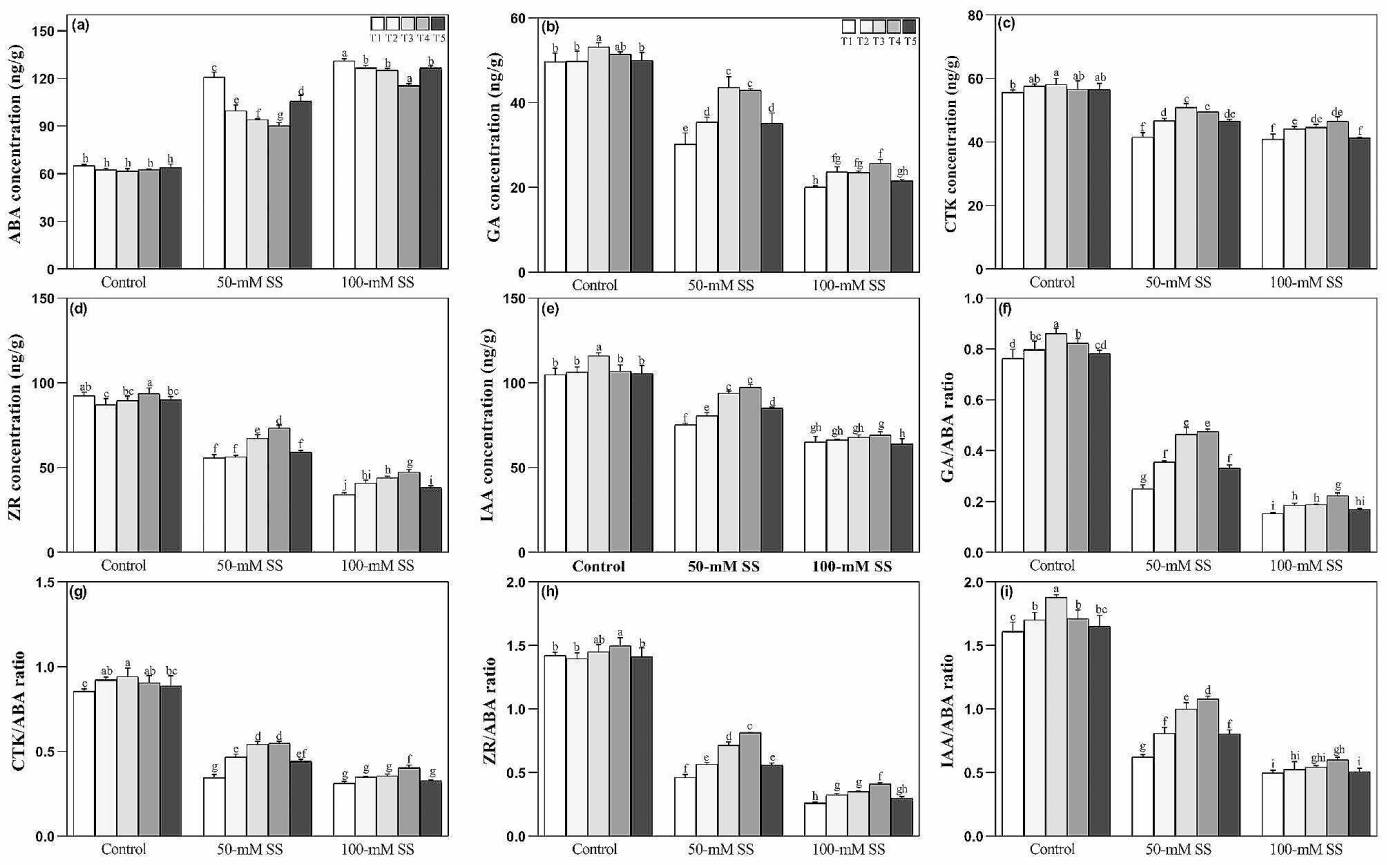
Effect of different ratios of NH_4_^+^:NO_3_^–^ ratio on (**a**) ABA (**b**) GA (**c**) CTK, (**d**) ZR, (**e**) IAA (**f**) GA/ABA (**g**) CTK/ABA, (h) ZR/ABA and (**i**) IAA/ABA in soybeans under controlled and saline stress conditions. Bars represent means ± SD (*n* = 3). Different letters indicate treatment differences at *p* < 0.05 (Duncan’s method)

In the present study, increased SS treatments significantly increased ABA but decreased growth hormones, including GA, CTK, ZR, and IAA, regardless of different ratios of AN application (Fig. 5a-e). Moreover, the ratios of growth-stimulating hormones (GA/ABA, CTK/ABA, ZR/ABA, and IAA/ABA) to ABA were downregulated under both stress levels, compared to the control (Fig. 5f-i). NH_4_^+^:NO_3_^–^ ratios reduced ABA but improved growth hormones and their ratios to ABA under both stress levels (Fig. 5a-i). Under both stress levels, ABA decreased at all other ratios compared to the higher NH_4_^+^:NO_3_^–^ ratio (T1). However, significantly lower ABA levels were recorded under T3 and T4 compared to T1, T2, and T5 in 50 mM stressed Seedlings and under T4 in 100 stressed seedlings (Fig. 5a). However, optimal AN ratios (T3 = 25/75 and T4 = 50/50) caused a significant increase in ZR under either stress (Fig. 5d) and GA, CTK, and IAA under 50-mM, compared to other ratios (Fig. 5b, c, e).

### Biochemical changes

**Fig. 6:**
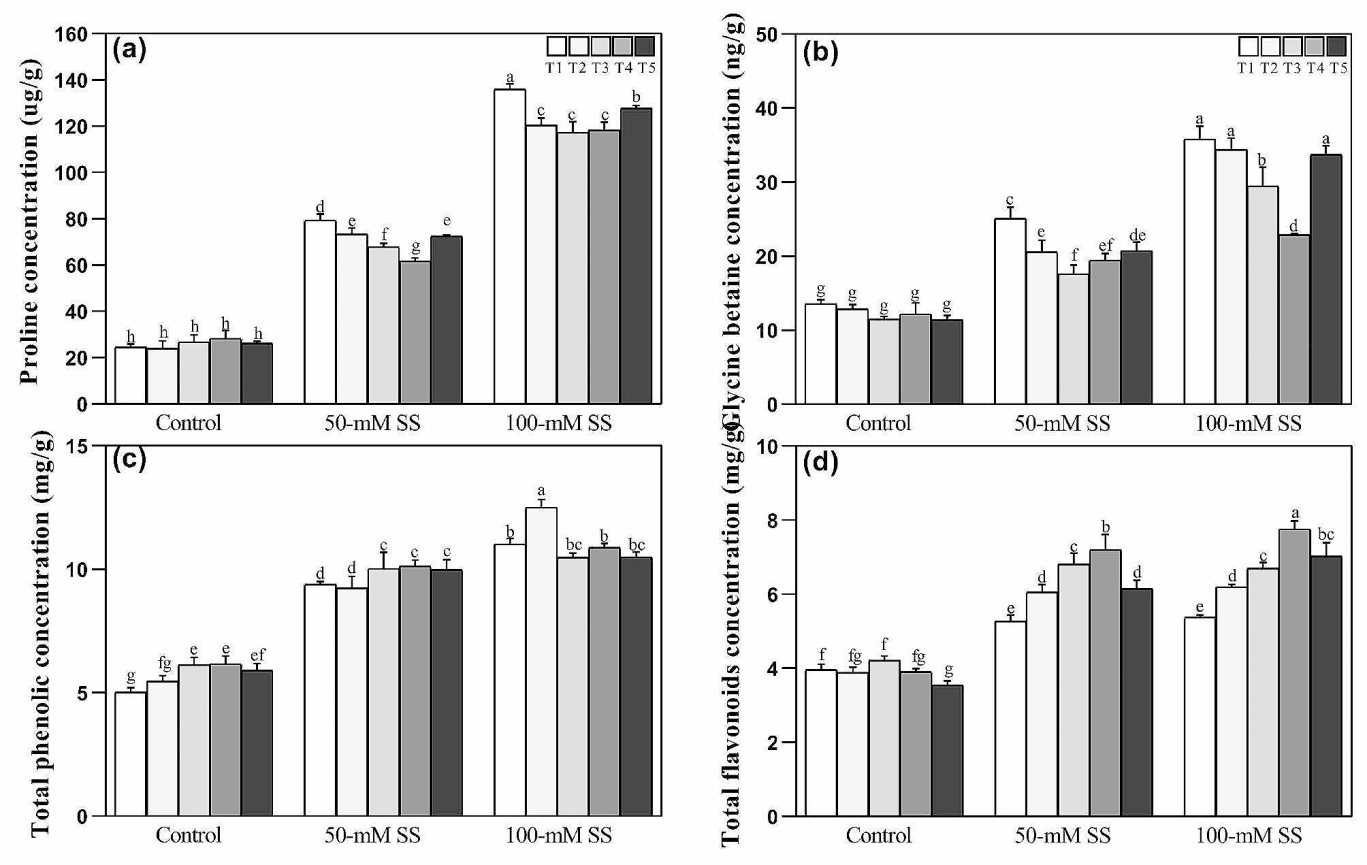
Effect of different ratios of NH_4_^+^:NO_3_^–^ ratio on (**a**) proline (**b**) glycine betaine (**c**) total phenolic, and (**d**) total flavonoids in soybeans under controlled and saline stress conditions. Bars represent means ± SD (*n* = 3). Different letters indicate treatment differences at *p* < 0.05 (Duncan’s method)

The concentration of proline, glycine betaine, total phenolic, and total flavonoid increased significantly in leaves of soybean seedlings under both SS levels compared to the control, regardless of NH_4_^+^:NO_3_^-^ applications (Fig. 6a-d). However, NH_4_^+^:NO_3_^–^ ratios, particularly optimal (25/100 and 50/50), significantly reduced the concentration of proline and glycine betaines under both stress levels, compared to higher NH_4_^+^:NO_3_^–^ ratio (100/0) (Fig. 6a, b). In contrast, saline-stressed seedlings displayed significantly higher total phenolic content at 50 mM and total flavonoid content at both stress levels when supplied with the optimal NH_4_^+^:NO_3_^–^ ratio (T3 and T4). (Fig. 6c, d).

### Relationship between the morpho-physio-biochemical parameters

**Fig. 7:**
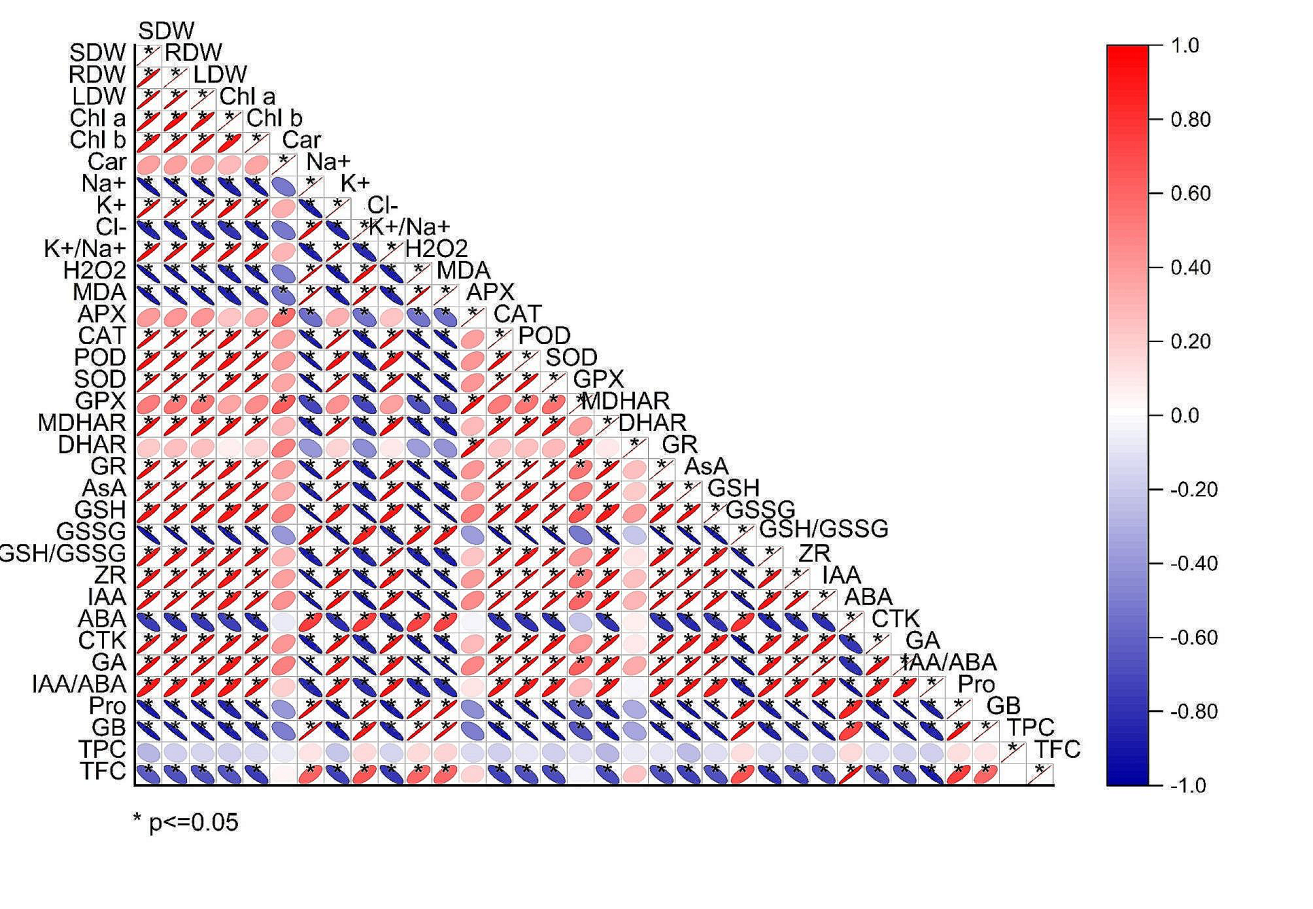
Associations between growth parameters and morphological responses. SDW: shoot dry weight; RDW: root dry weight; LDW: leaf dry weight; Chl a: chlorophyll a; Chl b: chlorophyll b; Car: carotenoids; ZR: zeatin riboside; GA: gibberellic acid; IAA: indole acetic acid; ABA: abscisic acid; CTK: cytokinin; AsA: ascorbate; GSH: glutathione; GSSG: oxidized glutathione; APX: ascorbate peroxidase; GR: glutathione reductase; MDHAR: monodehydroascorbate reductase; DHAR: dehydroascorbate reductase; MDA: malondialdehyde; H_2_O_2_: hydrogen peroxide; SOD: superoxide dismutase; POD: peroxidases; GPX: glutathione peroxidase; CAT: catalase; Pro: proline; GB: glycine betaine; TPC: total phenolic content; TFC: total flavonoids content; TFC: total flavonoids content. The matrix uses a color gradient ranging from blue (indicating strong negative correlations) to red (indicating strong positive correlations), with significant correlations (*p* ≤ 0.05) denoted by asterisks

In our study, we performed the pearson correlation matrix to represent the interrelationships among various physiological, biochemical, and growth parameters in soybean seedlings. Notably, shoot dry weight (SDW) exhibits strong positive correlations with root dry weight (RDW) and leaf dry weight (LDW), suggesting coordinated growth across different plant parts. Chlorophyll content (Chl a and Chl b) also positively correlates with SDW, highlighting the importance of photosynthetic pigments in growth. Oxidative stress markers, such as malondialdehyde (MDA) and hydrogen peroxide (H2O2), show significant positive correlations with antioxidant enzymes like catalase (CAT), peroxidase (POD), and superoxide dismutase (SOD), reflecting an integrated stress response mechanism. Furthermore, ion homeostasis parameters, such as the negative correlation between potassium (K^+^) and sodium (Na^+^), indicate stress-induced ion imbalance. Growth hormones, including abscisic acid (ABA) and gibberellins (GA), are correlated with several metabolic and stress-related parameters, underscoring their regulatory roles in stress adaptation. Proline (Pro) and other osmolytes demonstrate significant relationships with stress indicators, suggesting their function in osmotic balance and protection under stress conditions. Additionally, secondary metabolites like total phenolic content (TPC) and total flavonoid content (TFC) are interconnected with various physiological traits, indicating their role in plant defense mechanisms. This comprehensive correlation analysis underscores the complex interplay between growth, physiological processes, and stress responses, providing valuable insights into soybean seedlings strategies (Fig. 7).

### Principle component analysis

**Fig. 8:**
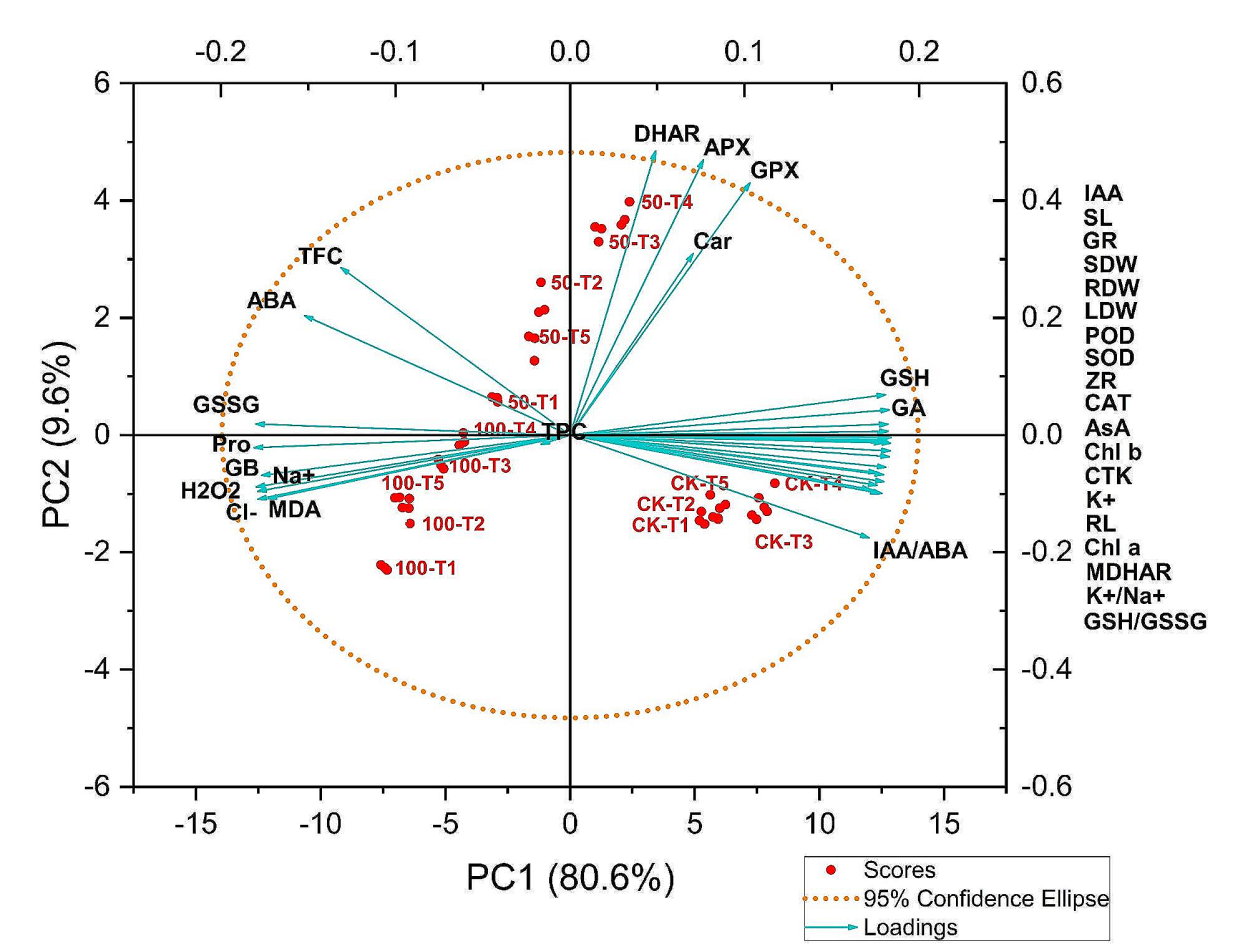
Principle component analysis (PCA) of growth and physiological responses. Chl a: chlorophyll a; Chl b: chlorophyll b; Car: carotenoids; ZR: zeatin riboside; GA: gibberellic acid; IAA: indole acetic acid; ABA: abscisic acid; CTK: cytokinin; AsA: ascorbate; GSH: glutathione; GSSG: oxidized glutathione; APX: ascorbate peroxidase; GR: glutathione reductase; MDHAR: monodehydroascorbate reductase; DHAR: dehydroascorbate reductase; MDA: malondialdehyde; H_2_O_2_: hydrogen peroxide; SOD: superoxide dismutase; POD: peroxidases; GPX: glutathione peroxidase; CAT: catalase; Pro: proline; GB: glycine betaine; TPC: total phenolic content; TFC: total flavonoids content

PCA analysis was conducted to determine the level of variability of the collected information and the correlation between the three salinity levels, i.e., 0 mM, 50 mM, and 100 mM, and the ammonium nitrate ratio and morpho-physiological attributes. As shown in Fig. 8, the two components (PC1 and PC2) accounted for 90.2% of the total variance in the data caused by the different treatments. 80.6% of the variation could be attributed to PC1, while 9.6% could be attributed to PC2. Using PCA, it was demonstrated that ammonium: nitrate ratios under controlled treatment had a substantial impact on biochemical indexes such as chlorophyll pigments (Chl a, Chl b), antioxidant enzymes (SOD, POD, and CAT), and phytohormones (ZR, IAA, and CTK). The biplot was primarily composed of three clusters. The oxidative stress indicators such as MDA, H_2_O_2_, GSSG, osmolytes (Pro, GB), and the accumulation of Na^+^ and Cl^−^ clustered together. In contrast, growth parameters (SL, RL, LDW, SDW, RDW), photosynthetic pigments (Chl-a, Chl-b), K^+^, K^+^/Na^+^, antioxidant enzymes (MDHAR, CAT, SOD, POD, GR), and hormones (IAA, ZR, CTK) clustered together, while enzymes involved in the ascorbate cycle (DHAR, APX, GPX) and Car were included in the group. According to the PCA plot, there is a positive correlation between plant growth, chlorophyll pigments, hormones, H_2_O_2_ scavenging enzymes, K^+^ ion, GPX, APX, and DHAR activity, GSSG, Pro, and GB, Cl^−^, Na^+^, MDA, and H_2_O_2_ content (Fig. 8). There was an unfavorable correlation between plant growth, chlorophyll pigments, hormones, enzymes that scavenge H_2_O_2_, and K^+^ ions, along with the oxidative stress parameters. It has been demonstrated that the antioxidant activity of the ascorbate-glutathione cycle (GPX, APX, and DHAR) reduces plant salinity stress. The ammonium: nitrate ratios (50-T3, 50-T4), however, had a significant effect on the DHAR, APX, GPX, and carotenoids under 50 mM saline stress, while 50-T1 had a significant impact on the TFC, ABA, and TPC. However, under higher salinity stress (100 mM), the 100-T4 ratio increased osmolytes accumulation (Pro, GB), indicating that the ammonium: nitrate ratios enhanced the tolerance to saline stress (Fig. 8).

## Discussion

**Fig. 9:**
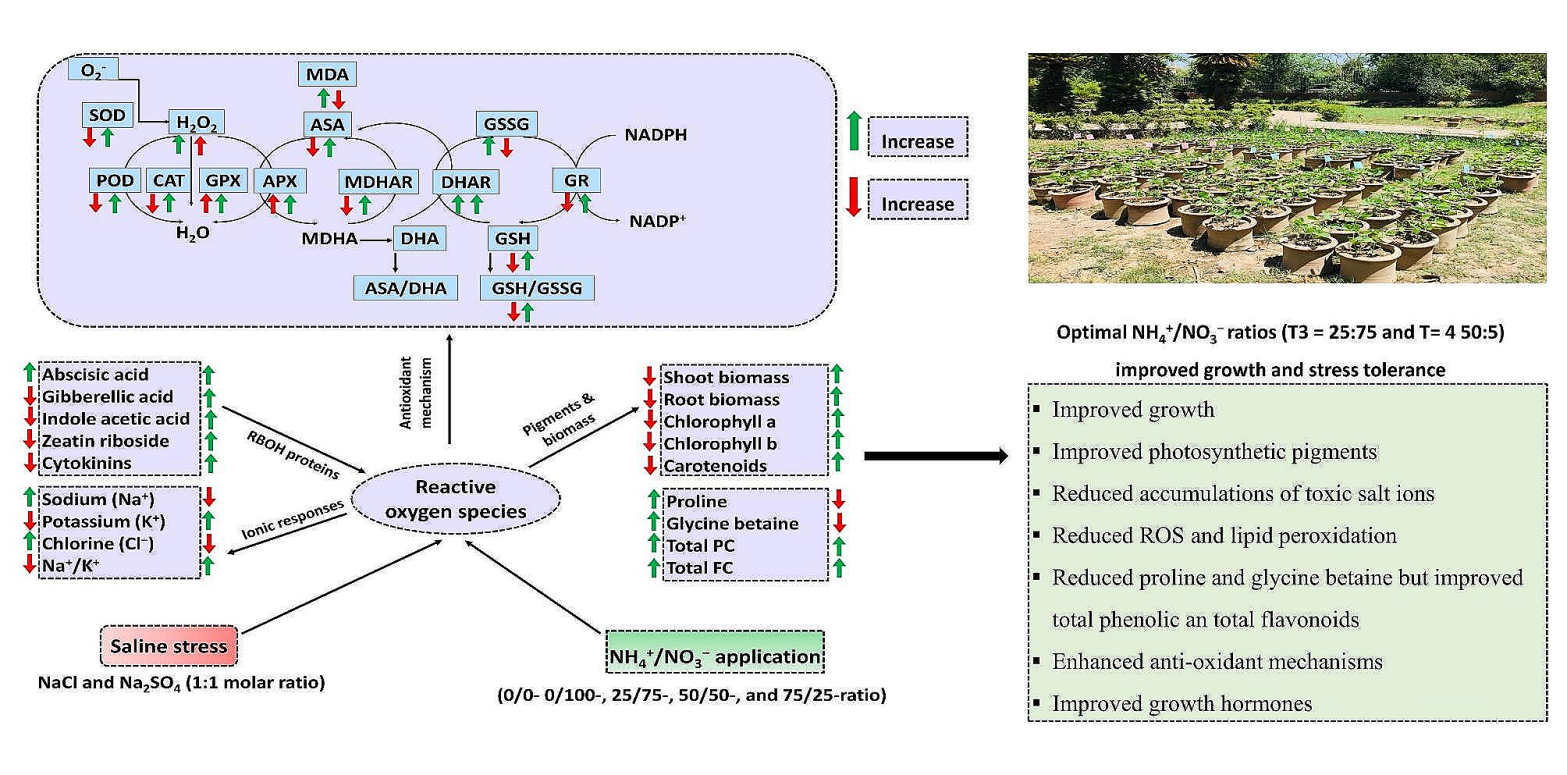
Schematic illustration of the morpho-physiological responses of soybean seedlings. SDW: shoot dry weight; RDW: root dry weight; LDW: leaf dry weight; Chl a: chlorophyll a; Chl b: chlorophyll b; Car: carotenoids; ZR: zeatin riboside; GA: gibberellic acid; IAA: indole acetic acid; ABA: abscisic acid; CTK: cytokinin; AsA: ascorbate; GSH: glutathione; GSSG: oxidized glutathione; APX: ascorbate peroxidase; GR: glutathione reductase; MDHAR: monodehydroascorbate reductase; DHAR: dehydroascorbate reductase; MDA: malondialdehyde; H_2_O_2_: hydrogen peroxide; SOD: superoxide dismutase; POD: peroxidases; GPX: glutathione peroxidase; CAT: catalase; Pro: proline; GB: glycine betaine; TPC: total phenolic content; TFC: total flavonoids content

In our study, both saline stress (SS; 50- and 100-mM) levels significantly reduced shoot length, root length, and dry weight of leaves, stem, and root, compared to the control, irrespective of applying different ratios of NH_4_^+^:NO_3_^−^. These are the commonly observed adverse effects of salinity stress on crop species [4, 6, 35, 36]. In plants, biomass production relies on cell division and enlargement, regulated by complex physiological, biochemical, and molecular responses. These processes, including salinity, are typically sensitive to abiotic stresses [37]. Soil salinity, for instance, induces oxidative damage and disrupts the balance of photosynthetic rate and photoassimilates crucial for new cell growth, consequently leading to biomass production [38]. Salinity-induced reductions in biomass production can also be attributed to plants allocating more energy and carbon resources to maintain salinity homeostasis rather than growth and development [7, 39, 40]. The higher accumulation of stress-alleviating metabolites, such as proline, glycine betaine, and other antioxidants observed in our study (Fig. 9), for example, diminishes the resources available to support growth [6, 41, 42]. Furthermore, salinity stress contributes to increased ion accumulation and oxidative stress, which damage the membrane of plants and compromise growth [17–19]. Therefore, the observed reduced growth in our study could be attributed to the increased accumulation of toxic salt ions, oxidative stress biomarkers, and impaired physiological response (Figs. 8 and 9).

However, NH_4_^+^:NO_3_^−^ ratios elicited a varied response in alleviating salinity toxicity, leading to improved shoot length, root length, and dry biomass of leaves, stems, and roots. Optimal nitrogen supplementation has been shown to enhance root nitrogen uptake [23], hydraulic conductance [43], growth hormones, protein synthesis, and structural carbohydrates, resulting in faster cell division, increased meristematic cell numbers, and biomass production [44]. In our study, seedlings supplied with different NH_4_^+^:NO_3_^−^ ratios under varying saline stress conditions displayed differential growth retardations, likely attributed to the interaction between NH_4_^+^ and NO_3_^−^ with salt ions (Na^+^ and Cl^−^). Under higher NH_4_^+^: NO_3_^−^ ratios, significantly reduced growth and biomass under saline stress might result from the antagonistic relationship of NH_4_^+^ with K^+^, which may partially counteract the benefits of NH_4_^+^ supplementation in improving salinity tolerance [23, 45, 46]. Additionally, saline stress resulted in a significantly higher accumulation of Cl^−^, a significant salt ion, which could also account for the relatively low salt resistance following the application of a higher NH_4_^+^:NO_3_^−^ ratio (T1; 100/0) [31].

Moreover, in our study, the observed significantly higher MDA concentration in NH_4_^+^-supplemented saline-stressed soybean seedlings suggests higher oxidative stress, contributing to lower salt resistance [23, 47, 48]. Nevertheless, the 0:100 ratio of NH_4_^+^:NO_3_^−^ (T2) also demonstrated a low potential to sustain plant growth under saline stress, possibly due to higher Na^+^ accumulation in cells [24]. Soybean seedlings supplemented with the optimal 25/75 and 50/50 ratios of NH_4_^+^:NO_3_^−^ (T3 and T4) exhibited better growth and resistance under saline stress, likely due to their potential role in inhibiting Na^+^ and Cl^–^ accumulation, promoting K^+^ uptake and K^+^/Na^+^ ratios [30], reducing oxidative stress and improving membrane stabilization [24], plant hydration and photosynthetic efficiency [23, 49, 50]. The chlorophyll a, b, and carotenoid concentration decreased significantly under both saline stress levels compared to the control, regardless of NH_4_^+^:NO_3_^−^ application. This could result from oxidative stress, lower pigment biosynthesis, or accelerated degradation due to higher chlorophyllase activity, all impairing pigment concentration, resulting in lower photosynthetic rate and plant growth [36, 51]. Photosystems primarily comprise nitrogen, and its availability improves photosynthetic pigment synthesis [52]. We observed that optimal NH_4_^+^:NO_3_^−^ ratios (T3 and T4) improved chlorophyll concentration under either stress, while chlorophyll b and carotenoid concentrations under 50-mM stress compared to their higher ratios, consistent with previous findings [53].

Higher MDA accumulation in cells under unfavorable conditions indicates plant oxidative stress damage. In our study, saline-stressed soybean seedlings displayed higher MDA concentration, regardless of NH_4_^+^:NO_3_^−^ supplementation, which was attributed to a significantly higher accumulation of H_2_O_2_ under both stress levels than the control. Excessive ROS accumulations degrade DNA, proteins, and lipids, resulting in cellular death [54]. They also degrade photosynthetic pigments and reduce net photosynthetic rate and biomass production. In plants, antioxidant enzymes and metabolites can scavenge ROS, reducing oxidative damage under salinity stress [55]. In addition, saline-stressed seedlings exhibited lower concentrations of AsA and GSH but higher GSSG concentration, resulting in an impaired AsA-GSH pool and their metabolizing enzymes, indicating the sensitivity of the AsA-GSH cycle. Stress conditions reduce ASA and GSH [56, 57], resulting in an impaired AsA-GSH cycle, which usually helps plants scavenge H_2_O_2_ and minimize oxidative stress.

Several studies have indicated that salt-tolerant species possess robust anti-oxidant mechanisms, which confer salt resistance and increase their chances of survival under saline stress, compared to species with weak anti-oxidant mechanisms [6, 58]. In our study, salt-treated soybeans displayed weakened O_2_^•−^-H_2_O_2_-scavenging enzymes such as SOD, CAT, POD, MDHAR, and GR under both stress levels while exhibiting reduced APX and DHAR under 100-mM stress, demonstrating their sensitivity to saline stress conditions. Many recent studies have reported that N-supplementation increases the anti-oxidant mechanisms in plants, resulting in lower ROS accumulation and reduced oxidative stress damage [23, 59, 60], which supports our findings.

In our study, soybean seedlings supplemented with the optimal ratios of NH_4_^+^:NO_3_^−^ (T3 = 25/75 and T4 = 50/50) exhibited the highest reduction in H_2_O_2_ and MDA concentrations, while enhanced antioxidant enzymes resulted in improved antioxidant mechanism. Recently, the optimal application of NH_4_^+^ and NO_3_^−^ has emerged as a potential strategy to enhance plants’ anti-oxidant potential against oxidative damage associated with abiotic stresses, including salinity [23, 30, 60, 61], which supports our findings. A significant improvement in physiological characteristics was observed through the optimal NH_4_^+^:NO_3_^−^ ratio, which inhibited salt ions and ion toxicity, membrane damage, and chlorophyll destruction: this enhanced membrane stability, cellular hydration, and photosynthesis [24, 49]. Additionally, the significant decline in MDA concentration under saline conditions due to optimal NH_4_^+^ and NO_3_^−^ application is explained by the optimization of AsA-GSH pools (non-enzymatic antioxidants) and metabolizing enzymes that remove H_2_O_2_, protecting membranes and photosystems from oxidative stress [62]. Moreover, optimal NH_4_^+^:NO_3_^−^ ratios also contributed to the tolerance mechanisms against saline stress by regulating ion levels, inhibiting Na^+^ and Cl^−^ concentrations, and enhancing the K + and the K+/Na + ratio concentration. This resulted in less membrane damage. It has been suggested that NH_4_^+^ interacts antagonistically with Na^+^ and NO_3_^−^ with Cl^–^, inhibiting the uptake of Na^+^ and Cl^–^ by soybeans. As a result, soybeans are equally susceptible to the toxic effects of Na^+^ and Cl^–^ [63].

Our study indicates that increasing saline stress levels significantly elevates the concentrations of proline, glycine betaine, phenols, and flavonoids, compared to the control, regardless of the NH_4_^+^:NO_3_^–^ application. Salinity-stressed soybeans appear to prioritize producing or utilizing non-antioxidant metabolites, which may contribute to osmotic regulation, energy metabolism, or other stress-responsive pathways [64, 65]. Plants under stress typically produce osmolytes like proline and glycine betaine, which protect proteins and membranes, regulate redox balance, and maintain ion homeostasis during stressful conditions [66]. However, NH_4_^+^:NO_3_^–^ ratios, particularly optimal ratios (25/100 and 50/50), significantly reduced the concentrations of proline and glycine betaines under both stress levels, compared to higher NH_4_^+^:NO_3_^–^ ratio (100/0). This reduction was attributed to lower oxidative damage observed under optimal NH_4_^+^:NO_3_^–^ ratios. Additionally, this optimization may reduce metabolic costs, allocating energy and resources to other processes [67], such as the upregulation of the enzymatic antioxidant mechanism or growth and development [8]. Furthermore, the optimal NH_4_^+^:NO_3_^–^ ratio increased total phenols under 50 mM stress and total flavonoids under both stress levels. In plants, phenols and flavonoids play significant roles in antioxidant mechanisms, scavenging ROS, plant defense, signaling, and mediating auxin transport [68, 69].

Hormones are crucial in enabling plants to withstand stress conditions [16]. During drought stress, plants typically show decreased concentrations of growth-promoting hormones (IAA, CTK, GA) and increased concentrations of stress hormones (ABA, JA, SA). In our study, saline-stressed soybeans exhibited higher concentrations of ABA but lower levels of IAA, GA, CTK, and ZR, resulting in a reduced ratio of these hormones to ABA (IAA/ABA, GA/ABA, CTK/ABA, and ZR/ABA), regardless of different NH_4_^+^ and NO_3_^–^ application ratios. The imbalance in phytohormones observed in our study was associated with a significant reduction in growth and biomass in soybean seedlings [70]. Each plant hormone serves diverse biological functions, playing complex roles across various stages, tissues, and environments. Under abiotic stress, hormonal regulation can mediate physiological and metabolic responses and enhance plant tolerance [71]. For instance, hormones can modulate oxidative stress responses by interacting with ROS mediated by respiratory burst oxidase homologs (RBOHs), leading to distinct transcriptomic and physiological cascades. Several studies have demonstrated that salinity increases ROS production RBOH activity, which in turn inhibits hormone synthesis, resulting in decreased hormone concentrations [72, 73] (Fig. 9). In our study, saline-stressed soybeans displayed elevated levels of ABA, an endogenous signal that regulates stress tolerance mechanisms, including stomatal closure to prevent water loss and modulation of antioxidant mechanisms to minimize oxidative damage, thus playing a crucial role in salt stress defense [74]. Additionally, GA has been demonstrated to alter the regulation of several genes in tomato plants under stress, resulting in decreased plant length and dry weight [75]. Moreover, the activity, synthesis, metabolism, and transport of IAA are also affected by the interaction of stress with other hormones [15]. Under stress conditions, CTK concentrations may either increase or decrease. CTK regulates cell division, supports apical meristem, and mediates several physiological responses that aid plants in adapting to rapid environmental changes [76]. Therefore, the decrease in the concentrations of growth hormones such as GA, IAA, and CTK under saline stress may be one of the factors contributing to the sensitivity and severe reduction in shoot and root biomass compared to the control.

Furthermore, the optimal NH_4_^+^:NO_3_^−^ ratios reduced the concentration of ABA but improved concentrations of GA, CTK, and IAA under 50-mM stress, along with increased ZR concentration under both stress levels, compared to higher ratios. These growth-promoting hormone increases under stress could be linked to taller plant height and increased biomass production in soybean seedlings supplied with optimal NH_4_^+^:NO_3_^−^ ratios. The ratios of growth hormones to ABA also increased under optimal NH_4_^+^:NO_3_^−^ applications, attributed to the reduction in ABA levels and the increase in growth hormones. Plant growth is regulated by balancing growth-promoting and inhibiting hormones [77]. ABA, typically considered a growth inhibitor, may contribute to the enhanced growth of soybeans under optimal NH_4_^+^:NO_3_^−^ ratios due to its reduced levels, resulting in higher ratios of GA-IAA-ZR-IAA to ABA [78]. Hormones play crucial roles in physiological regulation through synergistic interactions involving transporters, receptors, and interconnected networks [79]. As a result, the endogenous hormones’ interactions under optimal NH_4_^+^:NO_3_^−^ ratios are likely to play a vital role in regulating soybean growth under control and saline stress conditions.

## Conclusions

In conclusion, our investigation elucidated the sensitivity of soybean seedlings to saline stress, evident in diminished growth attributes, chlorophyll pigments, compromised O2^•–^-H_2_O_2_ scavenging mechanisms (SOD, POD, CAT, MDHAR, and GR), alongside reduced levels of AsA, GSH and reduced primary growth hormones (GA, IAA, CTK, and ZR) under both 50mM and 100mM saline stress levels compared to the control, irrespective of NH_4_^+^:NO_3_^−^ ratios. This response was attributed to elevated accumulations of salt ions (Na^+^, Cl^−^) and oxidative stress markers (H_2_O_2_, MDA, GSSG). Nonetheless, soybean seedlings exhibited heightened levels of ABA and antioxidant metabolites (proline, glycine betaine, total phenolics, and total flavonoids), indicating reliance on antioxidant metabolite accumulation to mitigate saline stress sensitivity. However, applying distinct NH_4_^+^:NO_3_^−^ ratios prompted diverse responses in alleviating saline stress toxicity. Notably, the application of 25/75 and 50:50 NH_4_^+^ and NO_3_^−^ ratios resulted in improved resistance to saline stress by reducing salt ion uptake, enhancing K^+^ and K^+^/Na^+^ ratios, photosynthetic pigments, antioxidants, and growth hormones while reducing oxidative stress as evidenced by lower H_2_O_2_ and MDA, resulting in improved growth. While osmolytes such as proline, glycine betaine, and ABA exhibited an increase under saline stress conditions, their levels were notably diminished under optimal NH_4_^+^:NO_3_^−^ ratios. This phenomenon suggests a strategic response of soybean seedlings to optimize metabolic costs, potentially reallocating resources towards the primary ROS scavenging mechanism. Hence, our findings indicate that applying the optimal ratios of NH_4_^+^:NO_3_^−^, (T3 and T4) may be an effective strategy to improve saline stress in seedlings of soybean in particular and other crop species in general for sustainable agricultural practices.

## Materials and methods

### Experimental conditions

This study was conducted at the Department of Botany at Islamia College Peshawar (34◦15 North latitude and 71◦42 East longitudes), Khyber Pakhtunkhwa, Pakistan. The average temperature ranges from 5 °C to 39 °C between January-February and June-July, while the average annual rainfall is approximately 513 mm. Soybean seeds (Swat-84 cultivar) were sterilized in a 0.1% magnesium chloride solution for 5 min and then washed five times with distilled water. Subsequently, the seeds were then sown in pots (diameter 15 cm) with a bottom hole (diameter of 2 cm), each filled with 5 kg soil (silt-loamy with a pH of 6.9, EC of 0.28 ds/m, and a bulk density of 1.55 g). The 12-day-old soybean seedlings were subjected to three different saline stress conditions supplied with varying NH_4_^+^: NO_3_^-^ ratios (total 5 mM). T1 = 5-mM NH_4_^+^: 0-mM NO_3_^−^, T2 = 0-mM NH_4_^+^: 5-mM NO_3_^−^, T3 = 1.25-mM NH_4_^+^: 3.75-mM NO_3_^−^, T4 = 2.5-mM NH_4_^+^: 2.5-mM NO_3_^−^, T5 = 3.75-mM NH_4_^+^: 1.25-mM NO_3_^−^. NH_4_(SO_4_)_2_ and KNO_3_ was used for the application of NH_4_^+^ and NO_3_^−^, respectively. The solution containing different ratios of NH_4_^+^:NO_3_^−^was applied to the pots of both control and saline-stressed seedlings. The initial NH_4_^+^: NO_3_^−^ treatment was applied to 2-week-old seedlings, with subsequent treatments administered at 5-day intervals, totaling four applications. Finally, 40-day-old seedlings were harvested for growth and physiological analysis. The harvested samples were frozen in liquid N for physiological analysis and stored at − 80 °C for further laboratory analysis.

### Determination of growth parameters

We measured the shoot and root length using measuring tape. An electric balance was used to calculate the fresh and dry weights of the stems, leaves, and roots. Plants were oven-dried for 30 min (105 °C) and then dried at 75 °C until a constant weight was achieved.

### Estimation of photosynthetic pigment concentration

Photosynthetic pigments were extracted from dried leaves (0.10 g) using 80% acetone and anhydrous ethanol. Carotenoids, chlorophyll a, and chlorophyll b were quantified, measuring the absorbance at 440 nm, 645 nm, and 663 nm using a spectrophotometer [80].

### Estimation of mineral elements

Dry leaf samples were ground and transferred to a centrifuge tube containing 4 ml of deionized water. After boiling and centrifugation (4000 rpm, 10 min), the supernatant was collected in tubes with increased volume to 10 ml. The supernatants were used to determine the content of Na + and K^+^ using an inductively coupled plasma atomic emission spectrometer (Prodigy, Leeman, U.S.A.).

### Oxidative stress indicators

Hydrogen peroxide (H_2_O_2_) was quantified by measuring the absorbance of the titanium-peroxide complex at 410 nm [81]. Samples (0.2 g) were homogenized in trichloroacetic acid (0.1%) in a cold bath and then centrifuged at 5000 g (10 min, four °C). The supernatants were extracted and added with ammonia and titanium reagents. The resulting precipitate was centrifuged at 10,000 × g (10 min) after five acetone washes and added 1 M H_2_SO_4_. The thiobarbituric acid (TBA) test was used to measure the malondialdehyde (MDA) levels [82]. Fresh leaf samples (0.5 g) were homogenized in a 5% trichloroacetic acid (TCA) solution, followed by centrifugation at 5,000 *× g* (10 min, 4^◦^C). The supernatants were mixed with 20% TCA and heated at 100 °C (15 min) before a second centrifugation at 5,000 *× g* (10 min). Absorbance readings at OD450, OD532, and OD600 nm were conducted using a spectrophotometer. Additionally, the concentrations of AsA and GSH were determined spectrophotometrically based on the absorbance at 530 nm and 412 nm, following the standard methods described by Huang et al. [83] and Yu et al. [84].

### Estimation of antioxidant enzyme activities

Fresh leaf samples, each 500 mg, were extracted in ice-cold potassium phosphate buffer (100 mM, pH 7.0) and PVP (1%). The mixture was centrifuged at four °C for 15 min at 12,000× *g*, and the collected material was used for the assay of SOD, CAT, APX, GR, DHAR, and MDHAR. Leaf samples were homogenized in a solution of 50 mM phosphate buffer (pH 7.8) and 0.1 mM EDTA-Na_2_O, and the homogenate was then centrifuged at 12,000 *× g* (5 min, four °C). The experiments were then conducted using the supernatant containing an enzyme extract. For SOD (E.C.1.15.1.1) determination, a reaction mixture was prepared 50 mM phosphate buffer (pH 7.8), 130 mM methionine, 75 mM nitro-blue tetrazolium (NBT), 2.0 mM riboflavin, and approximately 0.1 ml of enzyme extract [85]. The absorbance was read at 560 nm. Peroxidase (POD; EC 1.11. 1.7) was estimated by reading absorbance (470 nm) using a spectrophotometer. For catalase (CAT) estimation, a reaction mixture containing phosphate buffer (pH 6.0), 0.25% (v/v) guaiacol, and hydrogen peroxide with enzyme extract was prepared. Catalase activity was determined by measuring absorbance at 240 nm over one-minute intervals for three minutes [86].

An enzyme extract of APX (EC 1.11.1.11) was used to measure the enzyme’s activity in a reaction mixture containing phosphate buffer (50 mM, pH 7.0), hydrogen peroxide (1.0 mM), and L-ascorbic acid (0.25 mM) [87]. A spectrophotometer detected a rise in absorbance at 290 nm after ascorbate oxidation. MDHAR (EC 1.6.5.4) and DHAR (EC 2.5.1.1.8) activities were assayed using standard methods [88, 89]. Briefly, MDHAR activity was determined by monitoring the decrease in absorbance at 340 nm for 1 min as NADPH was oxidized, and then the result was calculated using an extinction coefficient of 6.2 mM^− 1^ cm^− 1^. DHAR activity was measured by monitoring the increase in absorbance at 265 nm for 1 min and then calculated using an extinction coefficient of 14 mM^− 1^ cm^− 1^. By measuring the decrease in absorbance at 340 nm as NADPH is oxidized and using an extinction coefficient for the calculation, GR (EC 1.6.4.2) activity was determined [90].

### Determination of endogenous phytohormone levels

Using previously established methods, we extracted and purified ABA, JA, CKT, IAA, and ZR from leaf samples [91, 92]. The reaction mixture was prepared and transferred to a centrifuge tube containing 10 ml, thoroughly mixed, sealed, and placed in a refrigerator overnight at four °C for extraction. Moreover, centrifugation was carried out at 5000 *× g* for 30 min at 4 °C. The supernatant and 1 ml of the precooled extract were added to the remaining residue and allowed to stand for two hours at 4 °C. After centrifugation, the supernatants were combined. Following a prewash with methanol, the supernatants were separated through a Chromosep C18 column (C18Sep-Park Cartridge, MA, USA). The filtrate was collected, and the column was successively washed with methanol (100%) and diethyl ether (100%). The filtrate was then passed through a 0.22-mm membrane and dried using a N blower to remove the methanol. For enzyme-linked immunosorbent assays (ELISAs), the hormones containing fractions were diluted in phosphate-buffered saline, 0.1% Tween, and 0.1% gelatin (pH 7.5). ELISAs were used to quantify the concentrations of ABA, JA, CKT, IAA, and ZR [93].

### Statistical analysis

A total of three replicate measurements were conducted. An analysis of variance (ANOVA) was performed using SPSS version 16.0 (Chicago, IL, USA). Differences between means were considered significant when the ANOVA Duncan test *P*-value was less than 0.05. The figure graphics were created using GraphPad Prism 8. Additionally, a Pearson correlation analysis was conducted utilizing OriginPro 2019 software (Origin Lab Corporation Northampton, Northampton, MA, USA) to analyze growth parameters, photosynthetic pigment, ion concentrations, osmolytes, nitrogen metabolism, reactive oxygen species production, and antioxidant mechanisms. Principle component analysis (PCA) among the variables was performed using OriginPro 2019 software. PCA allows the relationship between variables to be observed.

## Data Availability

This published article includes all the data generated or analyzed during this study.

## Abbreviations

SDW: shoot dry weight
RDW: root dry weight
LDW: leaf dry weight
Chl a: chlorophyll a
Chl b: chlorophyll b
Car: carotenoids
ZR: zeatin riboside
GA: gibberellic acid
IAA: indole acetic acid
ABA: abscisic acid
CTK: cytokinin
AsA: ascorbate
GSH: glutathione
GSSG: oxidized glutathione
APX: ascorbate peroxidase
GR: glutathione reductase
MDHAR: monodehydroascorbate reductase
DHAR: dehydroascorbate reductase
MDA: malondialdehyde
H_2_O_2_: hydrogen peroxide
SOD: superoxide dismutase
POD: peroxidases
GPX: glutathione peroxidase
CAT: catalase
Pro: proline
GB: glycine betaine
TPC: total phenolic content
TFC: total flavonoids content
